# Retrieval (N400) and integration (P600) in expectation-based comprehension

**DOI:** 10.1371/journal.pone.0257430

**Published:** 2021-09-28

**Authors:** Christoph Aurnhammer, Francesca Delogu, Miriam Schulz, Harm Brouwer, Matthew W. Crocker

**Affiliations:** Department of Language Science and Technology, Saarland University, Saarbrücken, Germany; Tohoku University, JAPAN

## Abstract

Expectation-based theories of language processing, such as Surprisal theory, are supported by evidence of anticipation effects in both behavioural and neurophysiological measures. Online measures of language processing, however, are known to be influenced by factors such as lexical association that are distinct from—but often confounded with—expectancy. An open question therefore is whether a specific locus of expectancy related effects can be established in neural and behavioral processing correlates. We address this question in an event-related potential experiment and a self-paced reading experiment that independently cross expectancy and lexical association in a context manipulation design. We find that event-related potentials reveal that the N400 is sensitive to both expectancy and lexical association, while the P600 is modulated only by expectancy. Reading times, in turn, reveal effects of both association and expectancy in the first spillover region, followed by effects of expectancy alone in the second spillover region. These findings are consistent with the Retrieval-Integration account of language comprehension, according to which lexical retrieval (N400) is facilitated for words that are both expected and associated, whereas integration difficulty (P600) will be greater for unexpected words alone. Further, an exploratory analysis suggests that the P600 is not merely sensitive to expectancy violations, but rather, that there is a continuous relation. Taken together, these results suggest that the P600, like reading times, may reflect a meaning-centric notion of Surprisal in language comprehension.

## Introduction

Theories of sentence comprehension have recently focused on expectation-based processing and the notion of Surprisal [1–5]. Surprisal theory posits that the cognitive effort induced by a word is proportional to its expectancy in context, and has been shown to account for a wide spectrum of behavioral processing phenomena [2, 4, 6–12]. Crucially, however, properties of words other than their expectancy, such as the association of a word with the preceding context [13], are known to also influence online indices of comprehension. Given the central role of expectancy in current theories and the linking hypothesis of Surprisal theory, an important open question is whether it is possible to identify processing correlates that are specifically sensitive to expectancy/Surprisal and insensitive to association, as well as the time-course of these neural and behavioral correlates.

In the electrophysiological domain expectancy related measures, such as Surprisal and cloze probability, have typically been linked to the N400 component [14–17], a negative voltage deflection peaking around 400 milliseconds post stimulus onset, the amplitude of which is inversely related to the expectedness of a word in context. The N400 is, however, sensitive to many other linguistic (and non-linguistic) factors beyond expectancy as well, such as frequency [18], orthographic neighbourhood size [19, 20], and lexical association [21]. As a consequence, many studies that have been interpreted as evidence for expectancy effects—based for example on manipulations of Cloze or n-gram probability—are confounded with simple association. For instance, in the sentence manipulation “He spread the warm bread with **socks**/**butter**” [22], the word “socks” is not only unexpected with regard to the meaning of the entire sentence, but it is also not related semantically. That is, “socks” is semantically unassociated to the prior context-words, irrespective of their compositional meaning as an utterance, whereas the other target word, “butter”, is both semantically expected and associated. As a consequence, the N400 has functionally been interpreted as reflecting semantic integration [23–25], lexical retrieval [13, 26–31], or both integration and retrieval on more recent “hybrid” accounts [32–34].

Another salient component of the event-related potential (ERP) signal is the P600, a positive going shift becoming apparent from around 600 milliseconds post stimulus onset, which has initially been identified as a component that is sensitive to structural processing. Theories of the P600 have associated it with the reanalysis of existing (morpho-)syntactic structure (e.g., [35–37]), with syntactic integration difficulty (e.g., [38, 39]), conflict monitoring/resolution [40–44], and more recently with semantic integration processes [26, 45].

The retrieval view of the N400 and the semantic integration account of the P600 are at the core of the Retrieval-Integration (RI) Theory of language comprehension [26, 45–47]. The RI account predicts these two components to be differentially affected by association and expectancy. That is, lexical retrieval is indexed by the N400. As a specific case of general memory retrieval, lexical retrieval is the process by which the meaning of a word is accessed in long term memory. As such, the sensitivity of the N400 to linguistic properties like frequency, orthographic neighbourhood size, as well as association and expectancy, is explained by the influence of these properties on the ease with which the word meanings are retrieved. In particular, words that are associated with prior context, or that are more expected given the unfolding utterance interpretation, are easier to retrieve from long-term memory. Integration, on the other hand, is linked to the P600. Integrative processing is conceptualized as the cognitive process that incorporates the meaning of a new word into a compositional representation of the meaning of the utterance as constructed so far. Crucially, this resultant meaning representation is assumed to provide the relevant contextual cues for the facilitated retrieval of potential upcoming word meanings.

A key strength of the account is therefore that it makes simultaneous predictions regarding effects in both components. In fact, the decomposition of language comprehension into retrieval and integration is made even more explicit in the computational instantiation of RI theory. In this model, retrieval is instantiated by the function

$$\begin{array}{c} retrieve(word\;form,\;utterance\;context)\mapsto word\;meaning\;[∼\text{N}400] \end{array}$$ (1)

which maps an incoming orthographic/acoustic *word form* onto a representation of *word meaning*, while taking the unfolding *utterance context*—the utterance meaning constructed prior to the current word—into account [48]. The output of this function serves as input to the function

$$\begin{array}{c} integrate(word\;meaning,\;utterance\;context)\mapsto utterance\;meaning\;[∼\text{P}600] \end{array}$$ (2)

which serves to integrate the retrieved *word meaning* into the unfolding *utterance context*, to produce an updated *utterance meaning*. While the *retrieve* and *integrate* functions, which underlie the N400 and the P600 component, respectively, may both be influenced by the overall expectancy of a word, this is for different reasons. In the case of the former, it is because the expectancy of an incoming word may facilitate retrieving its meaning from long-term memory, while in the case of the latter, it affects the effort involved in updating the unfolding utterance meaning representation with this retrieved meaning.

Indeed, the effort involved in updating utterance representations has been the focus of Surprisal theory. The original formalisation of Surprisal Theory focused on syntactic comprehension [2], and has been generalized as the relative entropy, or Kullback-Leibler Divergence [49], of a new probability distribution over syntactic analyses (operationalized as parse trees of a probabilistic context-free grammar) resulting from the current word, compared to the previous probability distribution [4]. In light of this characterization, one would thus expect structurally-induced Surprisal effects, i.e. syntactic integration difficulty, to be reflected in an increase in P600 amplitude [37, 50]. However, building upon the considerable evidence that the P600 also indexes semantic integration difficulty as predicted by the RI account, Venhuizen et al. [5] have recently proposed that the P600 component more broadly indexes comprehension-centric Surprisal—the negative log-probability of the utterance meaning representation after processing a word; that is, they propose that the P600 amplitude induced by an incoming word is proportional to how unlikely the interpretation is after processing this word, given the interpretation before encountering it. This Surprisal measure is influenced by both linguistic experience, as well as knowledge about the world [5]. As Brouwer et al. [48] point out, this view of the P600 as reflecting comprehension-centric Surprisal follows from the RI Theory. Just as syntactic models determine the likelihood of alternative analyses based on linguistic experience, the RI model recovers interpretations that reflect the distributional characteristics of the utterances it is exposed to [48].

The most recent instantiation of RI theory thus predicts the P600 component of the ERP signal, which indexes the amount of effort involved in updating the unfolding utterance meaning representation with the retrieved meaning of an incoming word, to be the locus that is specifically sensitive to expectancy/Surprisal effects [48] and insensitive to association effects. That is, integration effort is assumed to increase to the extent that the utterance meaning representation resulting from integrating this word meaning is semantically, pragmatically, or structurally unexpected, given the utterance meaning representation prior to integration. Given that the retrieval processes underlying the N400 are, among other factors, also sensitive to expectancy, previously reported N400 effects of Surprisal are unsurprising; that is, RI generally predicts both N400 (retrieval) and P600 (integration) amplitude to increase as a function of unexpectedness (although sufficient priming can eliminate the N400 effect even for unexpected words; see below). RI theory is thus in line with the linking of Surprisal to the N400 via retrieval (as also proposed by Frank et al. [16]). In sum, on the RI account, the P600, as an index of compositional, semantic, integrative processes, should therefore be sensitive primarily to the expectancy of a new word with regard to the current utterance meaning representation, and crucially, insensitive to association. Further, the RI account predicts the N400, as an index of lexical retrieval, to be sensitive to both lexical association and expectancy.

This raises the question of how we can test the prediction that the P600 is the component that is specifically sensitive to expectancy/Surprisal, while the N400 is sensitive to both association and expectancy. In the extreme case (“He spread his warm bread with **butter** / **socks**”), where the manipulations of lexical association and expectancy are completely overlapping, it is impossible to tease apart the contributions of lexical association and expectancy to the N400. At the other extreme, evidence comes from constellations in which expectation and association disagree; that is, when expectancy is low, but association is high—e.g., “De vos die op de stroper **joeg**…” (lit.: “The fox that on the poacher **hunted**” meaning that the fox hunted the poacher) relative to “De stroper die op de vos **joeg**…” (lit.: “the poacher that on the fox **hunted**…”) [44]—unexpected words result in N400 amplitudes similar to expected words, showing no difference in retrieval difficulty (cf. the ‘Semantic Illusion’ or ‘Semantic P600’ literature; e.g., see [26, 40, 43, 51] for reviews). Crucially, for both of these kinds of manipulations, P600-effects have been observed in response to the unexpected words (for an overview, see [26, 40, 43, 52]).

An open question, however, is how precisely association and expectancy combine in affecting N400 amplitude; that is, the picture that emerges from studies investigating the combination of association and expectancy in between these extremes is less clear. Some studies found that association has no influence when the sentence is incongruent [53–55]. Others, by contrast, found a stronger effect of association for incongruent targets, when presented to the right visual field (left hemisphere) [56]. Similarly, it was found that in syntactically correct but not meaningful sentences word associations do play a role for the N400 [57–59]. Further, a reduction in N400 amplitude was observed for event-related compared to event-unrelated contextually anomalous target words [60]. Indeed, arguments against the role of association in semantic violations contrast starkly with the results observed in the aforementioned literature in which high association eliminates an N400-effect for unexpected words (e.g., [51], where high association leads an otherwise contextually improbable target word to not increase N400 amplitude). Other studies focused on specific aspects like visual half field paradigms [56], individual differences [61], or late processing stage [55]. The existing literature thus paints an inconclusive picture of the influences of expectancy and lexical association on ERPs: On the one hand, some studies have found that lexical association effects are attenuated for incongruent target words, on the other hand some studies found that association is relevant even for these incongruent target words.

In order to assess how expectancy and lexical association affect retrieval and integration, we created an experimental design that crosses these stimulus properties, while aiming to minimise the confounding of expectancy and lexical association. To achieve this, we maximise the orthogonality of the two manipulations in a context manipulation design (Table 1) that manipulates strong (A+) and weak (A-) lexical association differentially by means of an intervening adverbial clause, for both expected (E+) and unexpected (E-) words. We manually constructed items with expected and unexpected target words by using main verbs that either do (“sharpened”) or do not (“ate”) take the target word (“axe”) as a semantically fitting and expected direct object. While this manipulation of expectancy necessarily covaries with lexical association (analogous to [22]), the additional—independent—manipulation of lexical association is achieved by using an intervening adverbial clause (“before he the wood stacked” / “before he the movie watched”). This adverbial clause contains words that either are or are not related to the target word, without changing the overall expectancy of the target word that is established by the main clause.

**Table 1:** Design. Example item crossing the factors expectancy (E+–) and lexical association (A+–). Literal translation given in italics.

(A) A+E+ Gestern schärfte der Holzfäller, bevor er das Holz stapelte, die **Axt**… (*Yesterday sharpened the lumberjack, before he the wood stacked, the axe*…)
(B) A–E+ Gestern schärfte der Holzfäller, bevor er den Film schaute, die **Axt**… (*Yesterday sharpened the lumberjack, before he the movie watched, the axe*…)
(C) A+E– Gestern aß der Holzfäller, bevor er das Holz stapelte, die **Axt**… (*Yesterday ate the lumberjack, before he the wood stacked, the axe*…)
(D) A–E– Gestern aß der Holzfäller, bevor er den Film schaute, die **Axt**… (*Yesterday ate the lumberjack, before he the movie watched, the axe*…)

Importantly, and unlike previous studies, the association manipulation is completely independent of the expectancy manipulation, such that there is no dependency between the manipulated adverbial clause and the target word. Further, we choose a particularly strong expectancy manipulation in the form of a selectional restriction violation. This allows us to assess if expected target words that are less associated to the context, nonetheless produce an increase in N400 amplitude relative to associated and expected ones, and conversely, whether unexpected but associated targets have attenuated N400 amplitude relative to unexpected and unassociated ones. Furthermore, this strong expectation violation is intended to maximise the observability of both N400 and P600 effects in the face of spatiotemporal component overlap. That is, as demonstrated by Delogu et al. [51, 62], because of spatiotemporal component overlap—the summation of, and potential cancellation of the scalp-recorded activity from different neural generators—expected integration effects on P600 amplitude may sometimes be attenuated by a large, preceding N400, thereby not yielding a reliable effect in the average waveforms (see [63] for discussion). In order to maximise inferences about P600 modulation it is therefore important to address such spatiotemporal component overlap in both analyses [64] and experimental designs [51, 62]. This strong expectation violation is thus intended to neutralise the effects of spatiotemporal component overlap, in which the large predicted N400 amplitude for unexpected targets might otherwise obscure the effect of our manipulation with regards to P600 amplitude.

The materials were presented in two experiments: an ERP study and a web-based self-paced reading (SPR) study. RI theory, as an integrated theory of both the N400 and the P600, predicts N400 effects of retrieval facilitation due to both lexical association (Condition A relative to B, and C to D) and expectancy (Condition A relative to C, and B to D). Crucially, for the P600, RI predicts only an effect of expectancy (again, Conditions A / B compared to C / D). The self-paced reading study was conducted to obtain behavioral correlates for the same items. Based on Surprisal theory, we predict clear effects of expectancy, which—under the RI account—should pattern with the P600. Additionally, we can assess whether there is any additional influence of association on reading times, and compare the relative influence of the two factors in the critical and spill-over regions. We will elaborate on the results based on the integrated predictions of RI theory for the N400, the P600, and reading times, and based on the individual predictions of other theories.

## Experiment 1: ERPs

### Method

This study was conducted with the approval of the Deutsche Gesellschaft für Sprachwissenschaft (DGfS).

#### Participants

Forty-nine participants from Saarland University took part in the experiment, nine of which were excluded due to excessive artefacts or to technical problems during recording. The final forty participants (mean age 23; SD: 2.96; age range 19-29; 6 male) were all right-handed, native speakers of German (12 early bilinguals). All participants had normal or corrected-to-normal vision and none of them reported any form of color blindness. They gave informed, written consent and were paid 20€ for taking part in the experiment.

#### Materials

We created 140 sentence quadruplets following the context-manipulation design exemplified in Table 1. To manipulate lexical association independently of expectancy, the target word (*axe*) was preceded by an adverbial clause containing lexical material that either was (“before he *stacked the wood*” in A & C) or was not (“before he *watched the movie*” in B & C) lexically associated to the target. In order to rule out an explanation of the resulting ERPs in terms of shallow processing [65] or good-enough representation [66, 67], adverbial clauses were created such that no structural or thematic dependency of the target word with the adverbial clause was supported. Further, the adverbial clauses did not allow for a role-reversal reading, i.e. there was no ambiguity about the correct assignment of agent and patient roles, in order to avoid so-called semantic illusion effects (see [26, 40, 43] for overviews). Unambiguous readings were ensured by the use of definite articles marked uniquely as nominative and accusative, respectively.

Expectancy, the second experimental factor, was manipulated by using a main clause verb that renders the target word either an expected (“*sharpened* the lumberjack … the **axe**” in A & B) or an unexpected direct object continuation (“*ate* the lumberjack … the **axe**” in C & D), given the selectional restrictions of the verb. To rule out any explanation of the observed ERP modulations in terms of syntactic processing difficulty, the target word and the main verb matched both grammatically and regarding the preferred subcategorisation frame of the verb. Further, we avoided verbs with a preference for object-drop. The resulting match or mismatch between the main clause verb (*eat*) and the target (*axe*) was thus purely selectional. We also avoided animacy violations, which have previously led to stronger P600 effects than other types of semantic violations [68]. Finally, to rule out interpretations of potentially observed P600 effects as reflecting prediction errors in unexpected targets [69–73], we selected main clause verbs that did not create high expectations for a specific object noun (as validated in the Cloze norming study reported on below).

Each item ended with additional material following the target word (e.g. “and chopped the logs” for our archetypal item) to avoid sentence-final wrap-up effects on the target (even though their importance has been discussed as largely overstated [74]). More importantly, this additional material allows us to detect potential spillover effects in the follow-up self-paced reading experiment reported in Section 3. The complete list of materials used in this and the follow-up experiment is made available in an online repository. We also included 120 filler sentences, part of which were adapted from another study [51]. Half of the fillers were plausible and half implausible, matching the proportion of expected and unexpected target words in the experimental sentences. The source and locus of the implausibility varied among the implausible fillers. A portion of the fillers included adverbial clauses with unexpected words that made the described scenario implausible in order to increase attention to the (always plausible) adverbial clause of the experimental items.

*Cloze norming*. In order to validate the expectancy manipulation achieved through our pre-selected *main verb*—*target word* pairs, we collected Cloze data for the experimental sentences in a web-based experiment. Forty-eight native speakers of German were recruited through Prolific Academic Ltd. [75] and compensated with 8€ per hour. Participants gave their consent by agreeing to the written study conditions. They were asked to complete the sentences presented up to, but not including, the article of the target noun. The experiment was implemented using the experimental software Ibex [76]. The sentences were divided into four lists according to a Latin square design such that each participant was presented with an equal amount of sentences in each of the four conditions, totalling to 140 trials per person. Participants could enter as many words as they wished, but were shown example items with simple *article*+*target* and *preposition*+*article*+*target* completions. The 140 experimental items were randomly interleaved with 70 filler sentences. For 12 items, the two unexpected conditions C/D produced high-Cloze completions (different from the targets), indicating that these sentential fragments were highly constraining towards predicting a specific lexical item. We changed the main clause verbs of these sentences to achieve more uniform Cloze profile, i.e. we avoided contexts for implausible items that raise expectations for a specific plausible word. These modified sentences were presented in a Cloze test with new participants. Based on the results of the Cloze test, we selected the final 120 experimental items in such a way that the difference in Cloze probability between expected and unexpected targets (i.e., A&B vs. C&D conditions) was maximized and the variability within high- and low-Cloze targets was reduced (i.e., A vs. B and C vs. D conditions). The Cloze probabilities of the target for the final set of items in the four conditions are presented in Table 2. The non-zero Cloze for unexpected targets resulted from a very conservative approach in which the target word was counted even if it occurred as part of a compound-noun or was produced in sentential positions other than the object of the main verb.

**Table 2:** Descriptive statistics. Descriptive statistics of the results of the Cloze (scale 0-1) and the association (scale 1-7) norming studies.

Condition	Mean	SD	Range	Mean	SD	Range
	Cloze Probability			Main verb–target Association		
A	0.67	0.23	0.17–1	6.25	0.81	2.27–7
B	0.64	0.23	0.17–1	1.65	0.84	1–5
C	0.008	0.025	0–0.17	6.25	0.81	2.27–7
D	0.008	0.028	0–0.17	1.65	0.84	1–5
	Noun–target Association			Verb–target Association		
A	6.29	0.82	1.9–7	3.23	1.59	1–7
B	2.09	1.01	1–5.7	1.87	0.94	1–5.4
C	6.29	0.82	1.9–7	3.23	1.59	1–7
D	2.09	1.01	1–5.7	1.87	0.94	1–5.4

*Association norming*. In a second, web-based validation study, we aimed to quantify the lexical association of the target words with the lexical material appearing in the preceding adverbial clause. To this end, we presented participants with word pairs and asked them to rate how strongly associated they were on a 1-7 scale (7 meaning *highly associated*). We presented participants with each content word in the adverbial clause (e.g., the noun and the verb in “who watched the movie”) and the target (“axe”). Since the expectancy manipulation is achieved by using a different main clause verb (“sharpen” vs. “eat”), we collected association ratings also for these verbs and the target. Note that participants only rated word pairs, but never saw their source sentences, nor did they know that the words would be appearing in a sentence together. Sixty native speakers of German recruited through Prolific Academic Ltd. took part in this study. They did not participate in any other experiments reported in this article and were compensated 11.50€ per hour. Participants gave their consent by agreeing to the written study conditions. The experiment was conducted using Ibex [76]. Stimuli were divided into six lists such that each participant saw only one of the context word–target pairs from each item, resulting in 120 trials per participant. Association ratings for the three word pairs are shown in Table 2. Words in the adverbial clause were more associated to the target in conditions A & C than in conditions B & D. The difference was stronger for the nouns than for the verbs of the adverbial clause. Association scores for the two main clause verbs also differed, such that expected targets were highly associated to the main verb compared to unexpected targets. Main verb-target association was strongly correlated with Cloze probability (see Table 3). To avoid multicollinearity problems in our statistical models, we did not include main verb association in our analyses.

**Table 3:** Correlations. Correlations between stimulus properties.

	Cloze Probability	Main Verb Association	Noun Association	Verb Association
Cloze Probability	1			
Main Verb Association	0.851	1		
Noun Association	0.029	0.008	1	
Verb Association	-0.005	0.001	0.467	1

#### Procedure

The Electroencephalogram (EEG) was recorded while participants were seated in a sound-proof, electromagnetically shielded and dimly lit chamber. Sentences were presented to the participants using rapid serial visual presentation (RSVP) in E-prime 2 [77]. Participants first practiced with six items, half of which were sentences containing unexpected words. After the practice session, the experiment was conducted in three blocks of 80 sentences each, presenting the items in pseudorandomized order, with breaks between the blocks. Participants pressed a button to start the trial and a fixation cross appeared in the center of the screen for 750 ms. Next, each word of the sentence was presented centrally for 350 ms with a 150 ms inter-stimulus interval. Participants were then asked to judge the plausibility of the sentence by pressing one of two buttons (mapping to *yes*/*no*). The position of the *correct* and *incorrect* buttons varied randomly in order to avoid motor preparation effects. The position of the *correct*/*incorrect* buttons was indicated by the position on the screen of the words *Yes* and *No*, which were highlighted in green and red respectively to make them more salient.

#### Electrophysiological recording and processing

The EEG was recorded by 26 active Ag/AgCl scalp electrodes, using the standard 10-20 system. During recording, FCz was used as online reference and AFz as ground. Data were digitized at a sampling rate of 500 Hz. Eye-movement artefacts were monitored through the electro-oculogram of two electrodes placed horizontally at the outer canthi of each eye and two electrodes placed vertically above and below the left eye. Impedances were kept below 5*k*Ω on scalp electrodes and below 10*k*Ω on eye electrodes. No online filtering was applied. The EEG was re-referenced offline to the average of the left and right mastoid electrodes and band-pass filtered between 0.01 and 30 Hz. Epochs starting 200 ms preceding the onset of the target word and lasting until 1200 ms following target onset were extracted from the EEG signal. Trials with ocular and muscular artefacts were excluded using a semi-automatic procedure. Baseline correction was performed on the 200 ms pre-stimulus interval.

#### Analysis

We analysed the data using a regression-based ERP estimation technique (rERPs, [78]). This technique allow us to replace each individual scalp-recorded voltage with a voltage estimate from a regression model that optimally combines the manipulated variables (e.g., Cloze probability and association) to explain the variance in the signal (see also [64]). Thus, applying this technique results in the decomposition of each observed scalp-recorded voltage into the contribution made by different experimentally manipulated factors. In the traditional rERP framework, one regression model is fitted for each time point, electrode, and subject. We apply a variation of this technique by replacing the n models fitted for n subjects at each electrode and time point with a single linear mixed effects model (LMER) at each electrode and time point (see [64], for discussion and [79–81], for prior work using this method). That is, rather than fitting one model for each subject, we fit only a single linear mixed model that captures per-subject variability as a random effect. As an extension, per-item variability can straightforwardly be modelled in the same regression equation, by introducing per-item random effects. Thus, the general model specification becomes

$$\begin{array}{c} Y_{si}=\beta_{0}+S_{0s}+I_{0i}+\left(\beta_{1}+S_{1s}+I_{1i}\right)X_{1si}+\epsilon_{si} \end{array}$$ (3)

where *S* and *I* refer to random effects for subjects and items, respectively. Random intercepts are represented by *S*_0*s*_ and *I*_0*i*_. For each predictor *X*, random slopes *S*_*s*_ and *I*_*i*_ will be computed. The *ϵ* term represents the residual error, i.e. the unexplained variance in the data.

This approach effectively distributes the multi-dimensionality of the dependent variable (in space and time) across separate statistical models, while the intra-experimental variability (across subjects and items) is modelled within each model. To distinguish this approach from the *rERP* technique described in [78], we label it *lmerERP*. In a nutshell, this approach allows us to (1) generate model-estimated ERP waveforms for each electrode and time sample and inspect them visually, (2) quantify the fit of the model to the data by inspecting the residual error, i.e., the difference between observed and estimated voltages across conditions (the closer this difference is to 0, the better the fit of the estimates to the observed voltages), (3) inspect model coefficients for each time sample and electrode, and (4) inspect effect sizes (z-values) and assess statistical significance on each time sample and electrode.

Data analysis was conducted using the MixedModels package for Julia [82]. The analyses were performed on data from the three midline electrodes Fz, Cz, and Pz and on the time samples between 200 ms prior to stimulus onset and 1200 ms following it. Continuous predictors were Cloze probabilities and association ratings (both noun-target and verb-target association, for nouns and verbs appearing in the adverbial clause) collected during pre-testing. Predictors were always included as fixed effects and as per-subject and per-item random slopes. Since predictors were z-standardized, the model coefficients represent the change in voltage associated with 1 standard deviation increase in the predictor, for each time sample and electrode. To make model interpretation more intuitive, we inverted the predictors, by multiplying each predictor with -1. This results in the coefficients sign matching the sign of the predicted ERP deflection.

Data analysis proceeded as follows. First, we aim to maximize the fit of the two manipulated factors individually. To do so, we assess the residuals on contrasts that differ only with respect to the predictor of interest. More specifically, Conditions A and C were used for isolating the effect of Cloze probability, as the adverbial clause is the same in these conditions and association scores are therefore constant. Conditions C and D were used to isolate the effect of association, as most items in these conditions resulted in zero Cloze probability. The data from each of these pairs of conditions is then analysed in a regression model including an intercept and the single predictor of interest (as well as a random intercept and slope for this same predictor). At this stage, the effect of different data predictor (such as log transformation) on model fit can also be investigated. Finally, the data from all trials in the four conditions are re-estimated in a regression model including all selected predictors. We report coefficients and corresponding z-values from this set of models. We also report the p-values for two time-windows of interest: 350-450 ms (N400 time window) and 600-800 ms (P600 time window). To correct for multiple comparisons by controlling the inflated false-discovery rates, we used the method illustrated by [83]. We corrected for the false discovery rate within all electrodes and time-samples, but separately for the two time windows.

### Results

#### Plausibility judgement task

Participants judged the plausibility of the four conditions as expected based on our experimental design. Specifically, Condition A was rated 90.3% (A+E+; SD = 8.1) plausible, Condition B 86.2% (A-E+; SD = 9.9) plausible, Condition C 80.4% (A+E-; SD = 14.3) implausible, and Condition D 85.5% (A-E-; SD = 12.5) implausible. Average reaction time in Condition A was 598 ms (A+E+; SD = 296), 639 ms in B (A-E+; SD = 267), 611 ms in C (A+E-; SD = 285), and 628 ms in D (A-E-; SD = 285). Means and standard deviations were computed from the per-subject and condition averages.

#### ERPs

**Fig 1:**
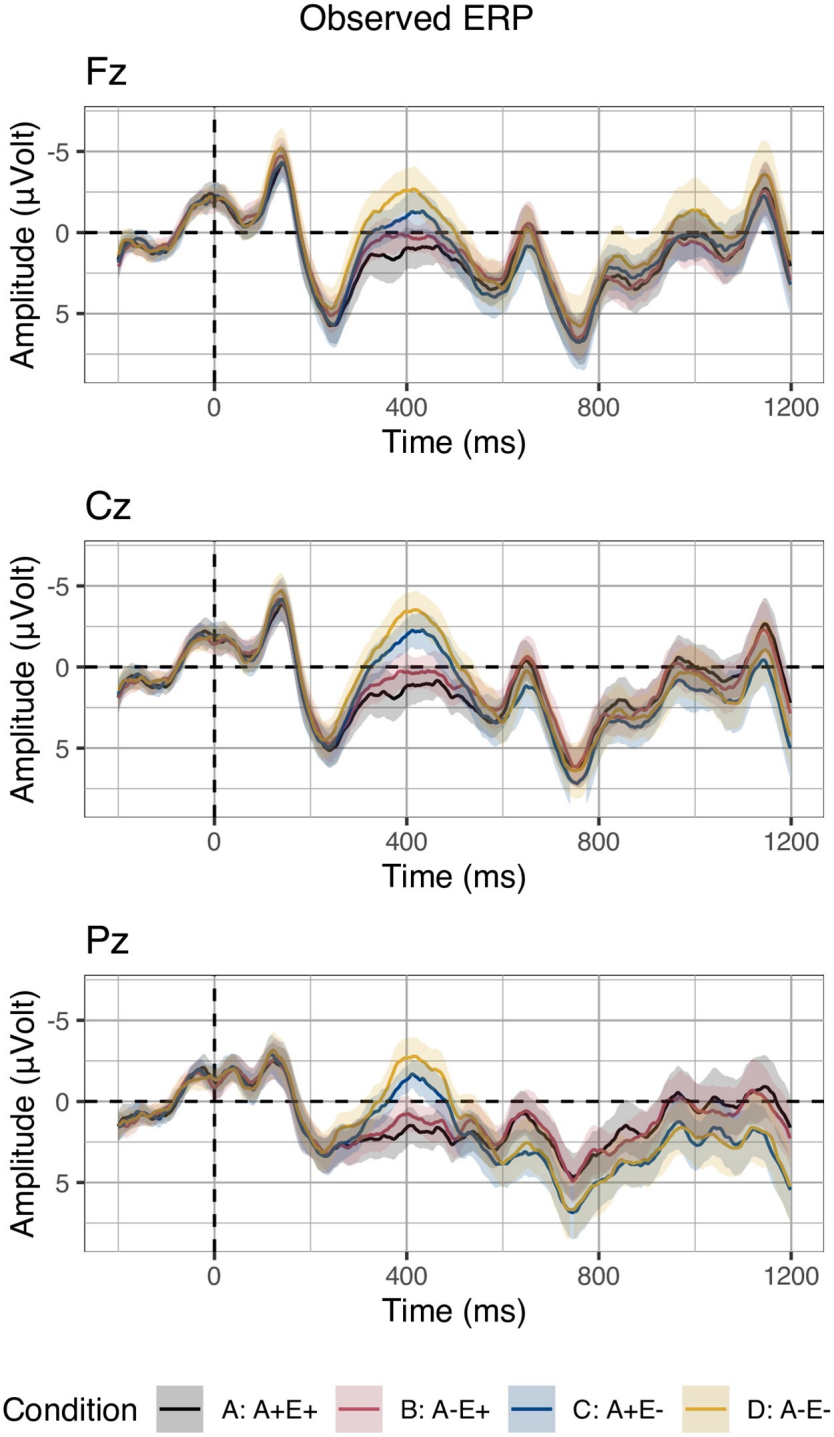
Grand-average ERPs. Grand-average ERPs on three midline electrodes in the four conditions crossing adverbial clause association and expectancy. Negative voltages are plotted upwards. Ribbons indicate standard error computed from the per-subject per-condition averages.

Fig 1 displays the grand average ERPs for the four experimental conditions on the three midline electrodes selected for analysis. Visual inspection suggests larger negativities in response to both less associated targets (conditions B relative to A and D relative to C) and unexpected targets (conditions C relative to A and D relative to B) in the N400 time window. In a later time window, approximately 600 ms post stimulus onset, a larger positivity appears in response to unexpected targets relative to expected ones on electrode Pz.

**Fig 2:**
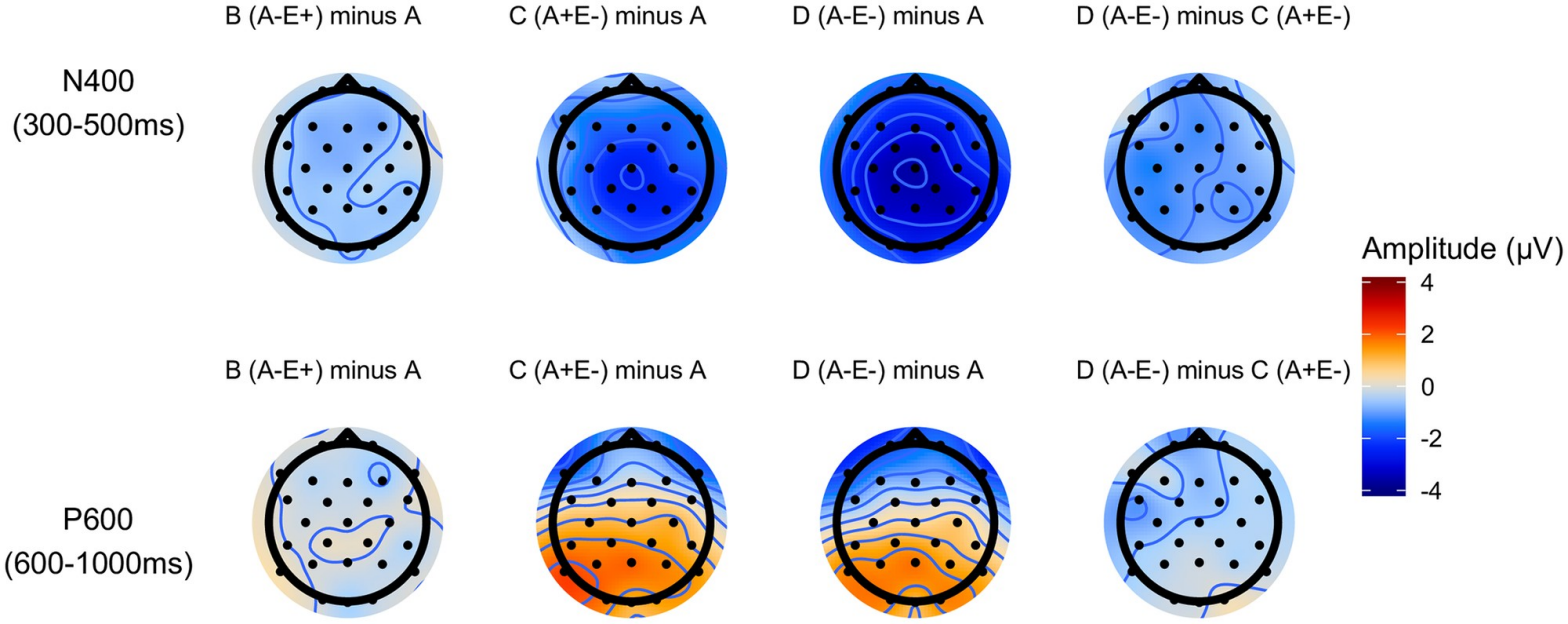
Scalp distributions. Topographic distributions of the average potentials in the N400 (row 1) and P600 time windows (row 2), relative to the baseline condition (columns 1-3) or relative to the unexpected-associated condition (column 4). Topographies computed from all non-reference electrodes.

Fig 2 shows the topographic distributions of the effects for each contrast of interest in the N400 and P600 time windows. In the N400 time-window, unexpected targets elicited a larger negativity compared to the baseline Condition A. A weaker N400 effect is also elicited by unassociated targets, within both expected and unexpected trials. The largest effect is observed for targets that are both unexpected and weakly associated. Between 600 and 800ms we observed a posteriorly distributed positivity, stronger over the left hemisphere, for unexpected targets compared to expected targets. A small negativity appears over the left fronto-central region for unexpected-unassociated compared to unexpected-associated targets (D relative to C), seemingly extending from the N400 time window.

**Fig 3:**
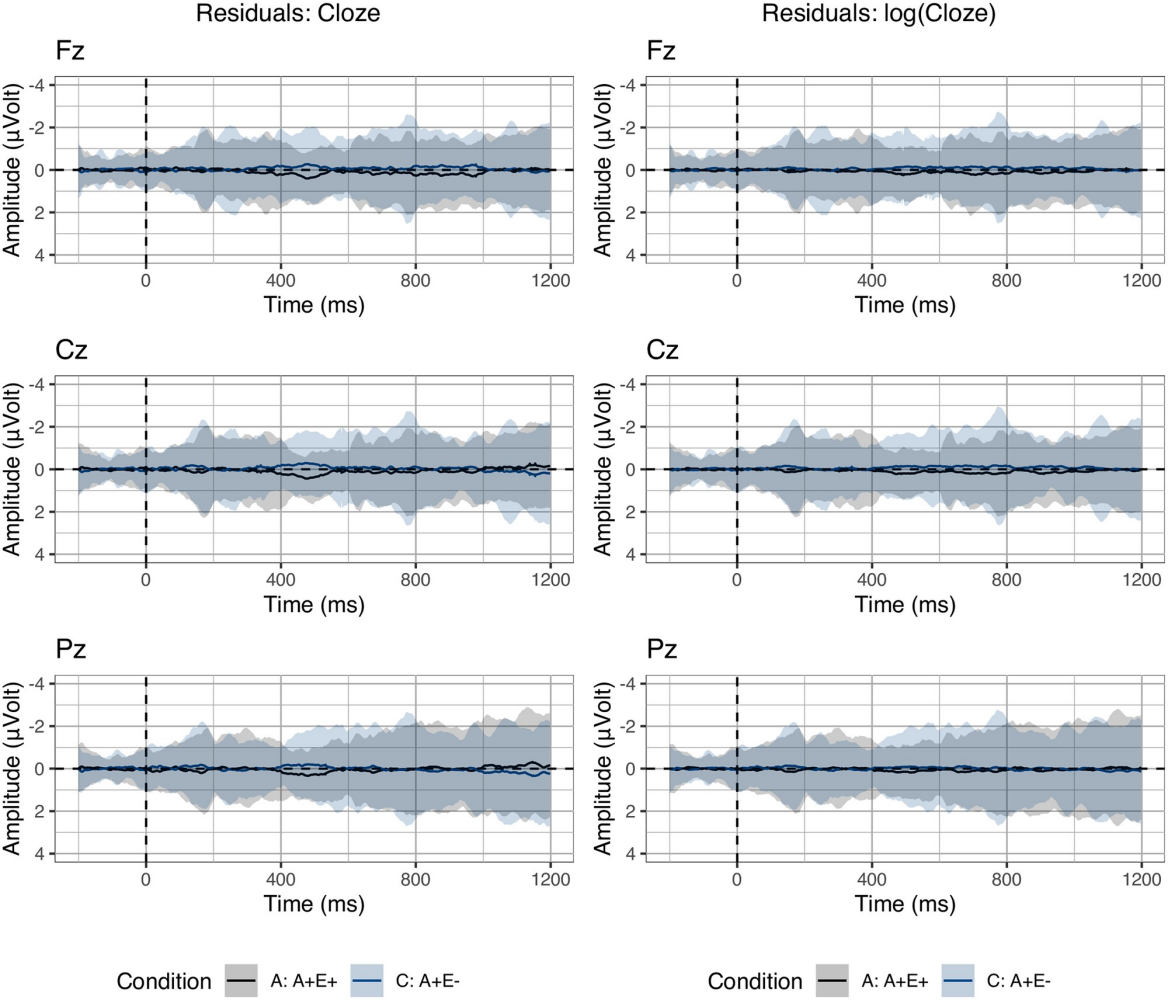
Residual error: Cloze. Residual error between observed voltages and estimated voltages in Conditions A and C using raw Cloze (left) or log(Cloze) (right) as predictor. Larger deviations from zero indicate larger model error. Ribbons indicate standard error computed from the per-subject per-condition averages.

To perform the lmerERP analyses, we first considered the single predictors individually (i.e., Cloze, noun-target association, and verb-target association) and assessed how well they fit the data as shown by the residuals (see Analysis section). To evaluate the fit of the Cloze probability predictor, we considered data from Conditions A and C. The residuals for the model including raw Cloze probability are shown in Fig 3 (left). Fig 3 (right) shows the residuals for the log-transformed Cloze probability (after smoothing Cloze by adding 0.01 to the Cloze values). We observed that log-tranformed Cloze probability visibly improves the fit compared to raw Cloze probability.

**Fig 4:**
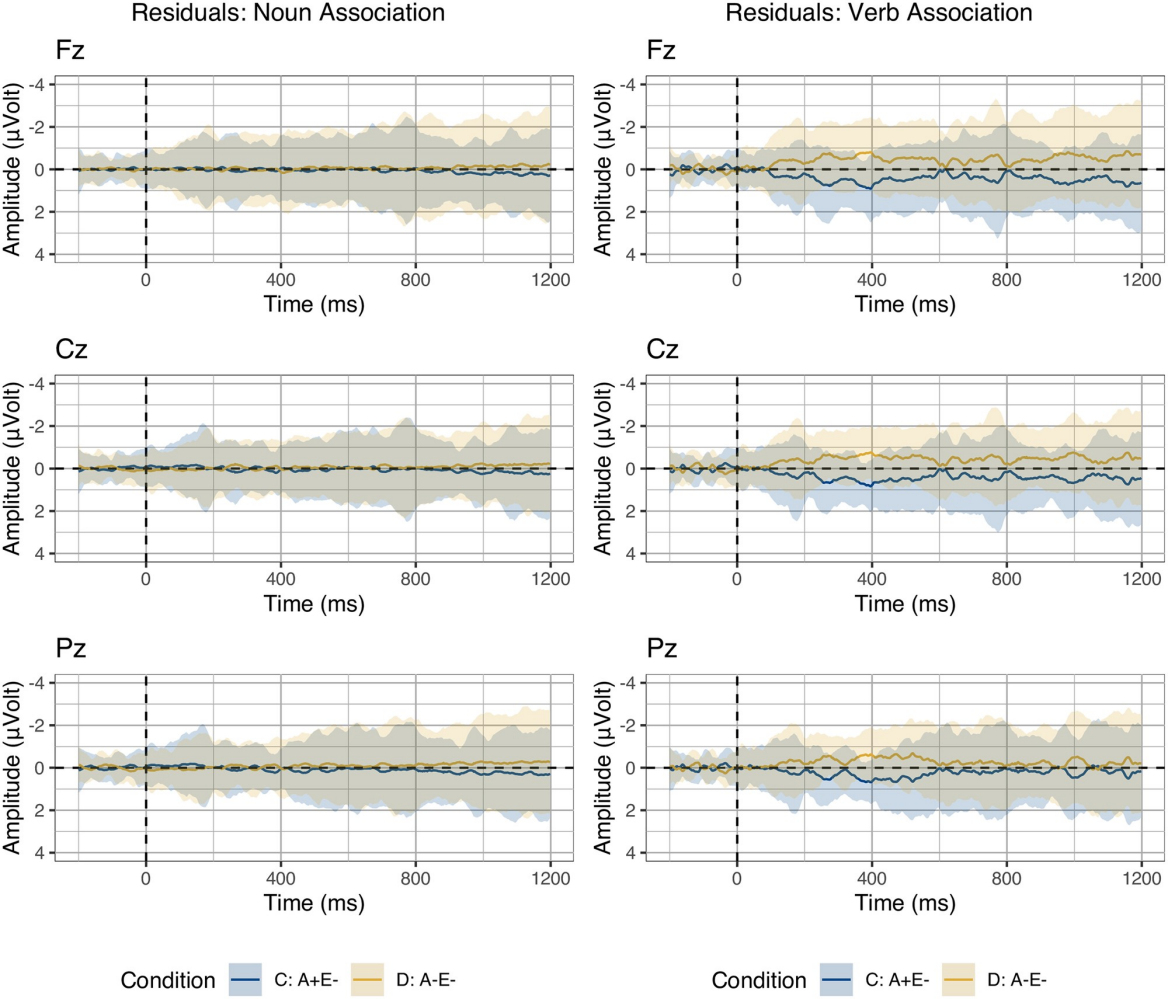
Residual error: Association. Residual error between observed voltages and estimated voltages in Conditions C and D using noun-target (left) or verb-target association (right) as predictor. Larger deviations from zero indicate larger model error. Ribbons indicate standard error computed from the per-subject per-condition averages.

To assess the fit of the association metrics, we considered data from conditions C and D, in which variability in Cloze is minimized as most items resulted in zero Cloze probability. For these metrics, no standard (non)linear transformation improved the fit compared to raw association values when inspecting the residuals visually. The residuals for the noun-target association and the verb-target association are shown in Fig 4. Noun-target association explains most of the variability in conditions C and D, nearly predicting their averages perfectly. We observed that adding verb-target association to a model that includes noun-target association did not improve the overall fit. We validated this finding by computing the mean of Akaike’s Information Criterion (AIC) values and the mean of the Bayesian Information Criterion (BIC) across models. These criteria of model quality take into account the model degrees of freedom, effectively penalizing the ones with a larger number of predictors (including random factors). Both BIC and (the less strongly penalising) AIC were lower (indicating better model quality) for models including only noun-target association compared to models including both noun-target and verb-target association values (AIC: 15816 < 15826; BIC: 15866 < 15916).

**Fig 5:**
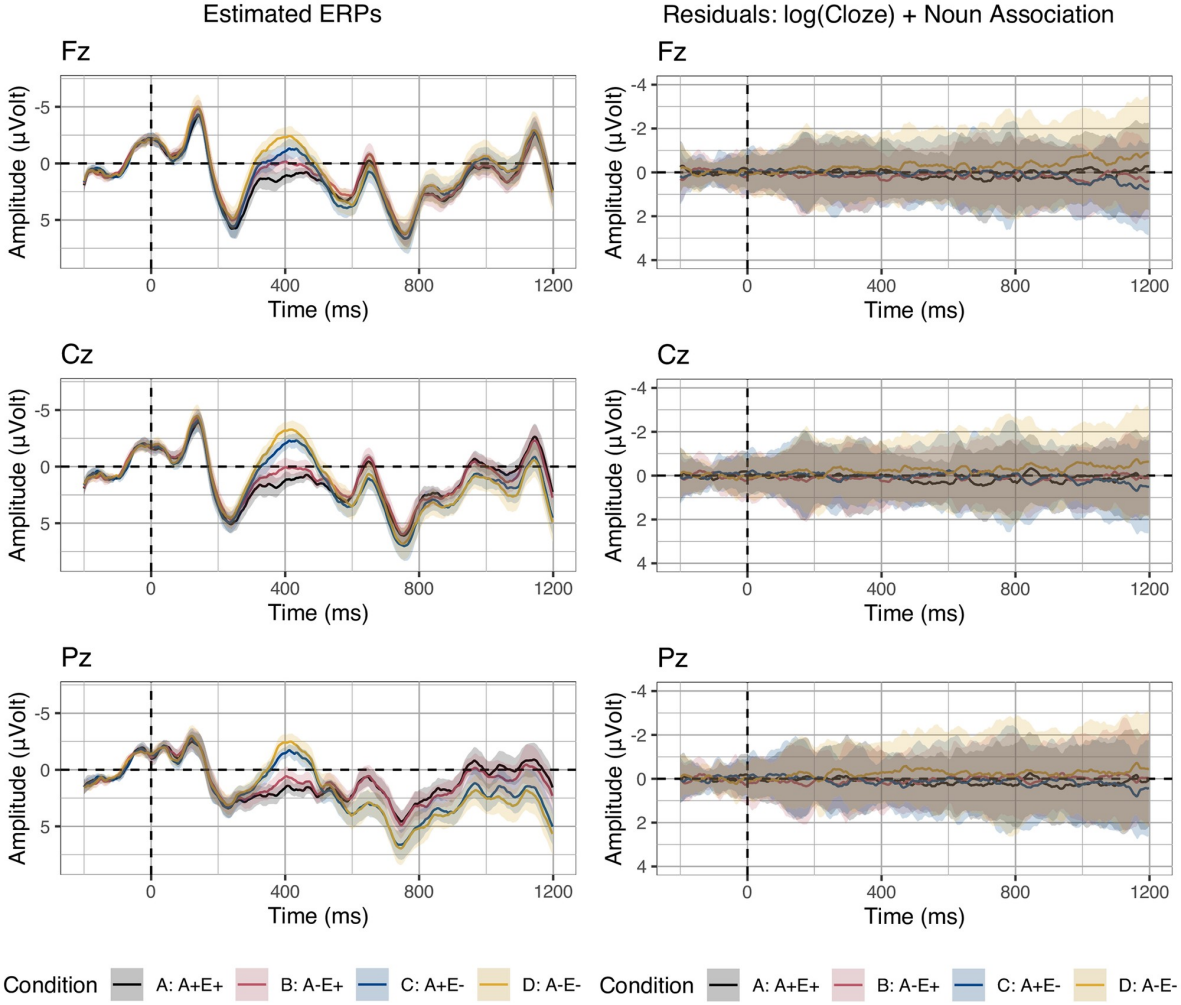
Estimated ERPs and residual error. Estimated ERP waveforms (left) and residual error (right) computed from lmerERP models with log(Cloze) and noun-target association as predictor. Ribbons indicate standard error computed from the per-subject per-condition averages.

Based on the results of the assessment of the individual predictors, we re-estimated the entire data set using log(Cloze) probability and noun-target association as predictors in an lmerERP analysis. The estimated ERPs and the residual error relative to the observed data is shown in Fig 5. The re-estimated waveforms show the same patterns as the observed data, namely a modulation of the N400 amplitude for both association and expectancy and a P600 effect in response to unexpected relative to expected targets. The residual error suggests that, on average, the N400 is underestimated for Condition D on electrode Pz. Furthermore, larger error is present in the very late portion of the epoch (approximately between 900 and 1200 ms).

**Fig 6:**
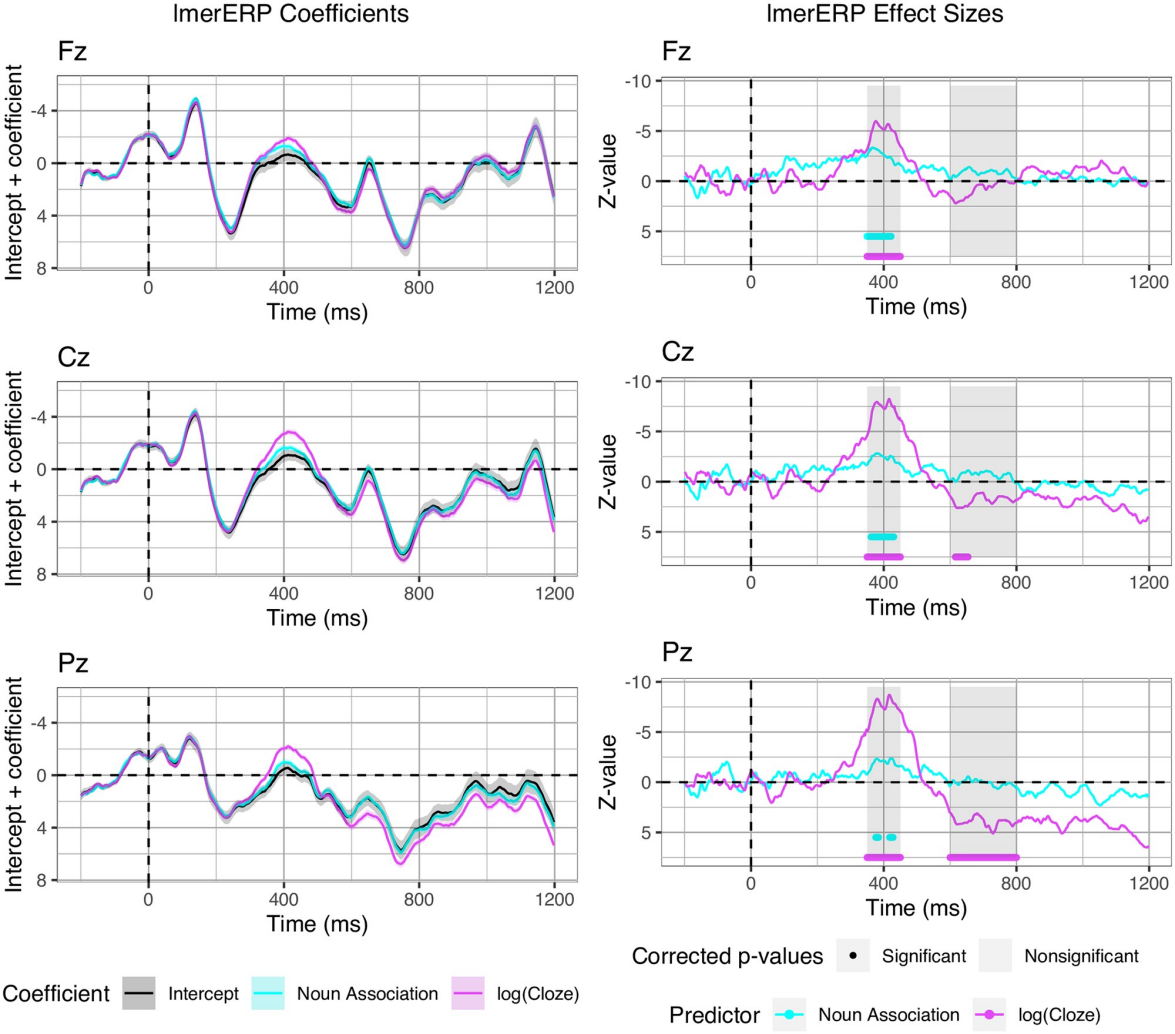
ERP coefficients and z-values. Coefficients (left; added to their intercept), effect sizes (z-values) and corrected p-values (right) from the lmerERP model with log(Cloze) and noun-target association as predictors. Ribbons indicate the standard error on the coefficients from the statistical model.

The coefficients from the model including log(Cloze) and noun-target association as predictors confirmed these observations, that is, a contribution of both log(Cloze) and noun-target association in predicting N400 amplitude while the posterior positivity in electrode Pz appears to be driven by log(Cloze) alone (Fig 6, left). The right hand graph of Fig 6 shows the corresponding z-values and the dots underneath the graph indicate statistically significant samples after multiple comparisons correction based on the false discovery rate. In the N400 time window there were significant contributions of log(Cloze) and noun-target association on all midline electrodes. The effect of noun-target association appears stronger on the frontal electrode Fz.

In the P600 time window, there was a significant effect of log(Cloze) in the posterior electrode Pz, and a smaller effect in the central electrode Cz. Beyond significance, the lmerERP analysis clearly showed that the predictors log(Cloze) and noun-target association can recover the observed N400 and P600 complex from the original data.

### Discussion

In Experiment 1, we investigated the effects of lexical association and expectancy on the N400 and P600 components of the ERP signal. Specifically, we examined whether it is possible to identify a specific locus of expectancy effects, insensitive to lexical association. We found that while both association and expectancy contribute to modulate the amplitude of the N400, the P600 was sensitive to expectancy alone.

In the N400 time window, words that were unexpected given the selectional restrictions of the main clause verb elicited larger N400 amplitudes than more expected targets, replicating previous findings (e.g., [16, 17, 22]). This effect was attenuated when the critical word was semantically related to lexical material appearing in the preceding adverbial clause, again replicating previous findings (e.g., [17, 57, 58, 60, 84]). What is interesting is that the influence of association on the amplitude of the N400 was not limited to anomalous targets, but was also present for congruent ones, with larger N400 amplitude for unassociated but expected targets relative to associated and expected ones (see also [79]).

In the P600 time window, unexpected targets elicited a larger P600 than expected targets in centro-parietal electrodes, while association had no effect. This finding is consistent with previous studies showing P600 effects elicited by semantic and world knowledge violations (e.g., [41, 44, 51, 52, 85–87]). Since in most of those studies, as well as in ours, expectancy was manipulated via a violation of a verb’s selectional restrictions, it is unclear if the observed P600 effects reflect expectancy or rather the detection of a semantic anomaly. To address this question, we subjected the ERP data to an additional exploratory analysis, in which lmerERPs were fitted to the EEG data recorded for Condition A only. This condition displays expected, non-anomalous targets that nonetheless exhibit variation in Cloze probability (ranging from 0.17 to 1). The main goal of this analysis was to assess whether Cloze probability in non-violating items predicts graded P600 amplitude on a trial-by-trial basis. This would provide evidence that the P600 is not sensitive only to categorical violations of expectancy, but rather a continuous correlate of word expectancy.

**Fig 7:**
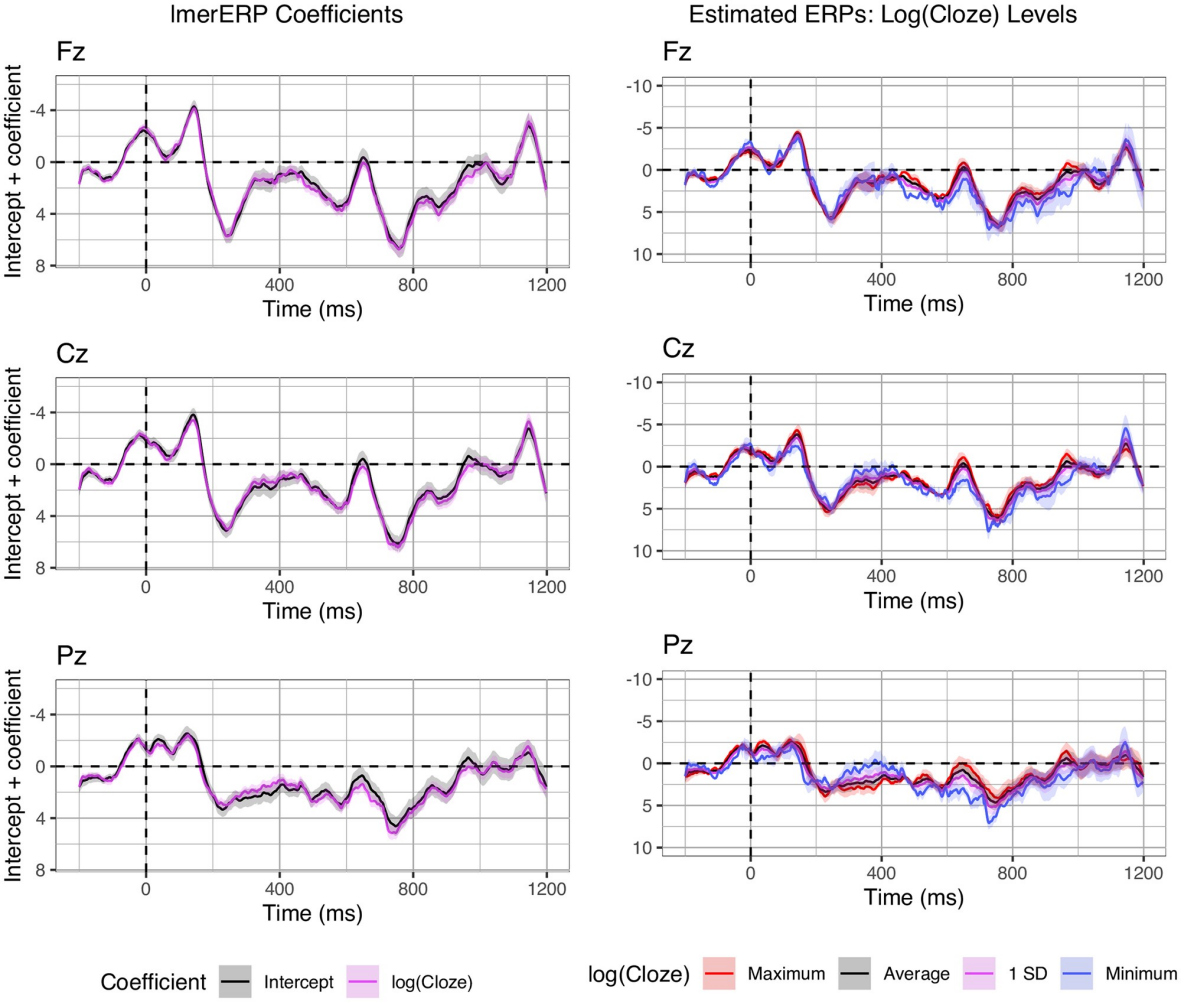
Exploratory analysis. Coefficients (left; added to their intercept) and estimated ERPs (right) for exploratory LMER models fitted only on Condition A. Error bars indicate the standard error on the coefficients from the statistical model (right) and standard error computed from the per-subject per-value averages (right).

As this analysis is conducted post-hoc and the stimuli are not explicitly designed to investigate graded effects of Cloze probability, the results are to be interpreted with appropriate caution. We focus our analyses on the coefficients to assess when (in which time-samples), where (in which electrodes) and to what extent (amplitude) log(Cloze) probability predicts voltage deviations from the intercept. As can be seen in Fig 7 (left), the coefficients appear to suggest a biphasic N400-P600 pattern in electrode Pz. Since we use z-standardized predictors, the coefficients are mathematically equivalent to the estimated waveforms at average log(Cloze) probability (intercept) and at 1 standard deviation below average log(Cloze) probability (see also [80]). Accordingly, Fig 7 (right) displays the estimated waveforms for the entire range of log(Cloze) probabilities for Condition A, i.e. including the minimum and maximum values (cf. Table 2). None of the corresponding z-values reached significance in this subset of only one-fourth of the original data.

## Experiment 2: Self-paced reading

Experiment 1 provides evidence that the P600 is specifically sensitive to expectancy and insensitive to association, while both expectancy and semantic association contribute to modulation of the amplitude of the N400. In Experiment 2, we examine the relationship between these effects and behavioral processing measures. Previous work has shown that Surprisal as estimated from language models accounts for a wide spectrum of behavioral processing phenomena, including reading times (e.g., [2, 4, 7, 9, 12, 14, 88]). These studies, however, were not explicitly designed to examine the influence of both association and expectancy on online processing. Eye-tracking studies, investigating how association and plausibility interact in discourse, found robust effects of plausibility, while the effect of lexical association was weaker and appeared to be modulated by the global context (see [89]). For example, Camblin et al. [55] showed robust effects of plausibility on eye-movements, while lexical association had a smaller and more localized effect, and only on incongruent words. Similar results were found by Brouwer et al. [48] in a self-paced reading study showing a significant effect of plausibility, but not of association. Thus, it is not clear to what extent behavioral measures may capture the N400 effects of association that we observed in Experiment 1, beyond the effects of expectancy. Moreover, Frank [90] has argued that any effect of semantic relatedness on reading times may be due to a confound with word predictability. We therefore conducted a self-paced reading experiment using exactly the same stimuli as those used in the Experiment 1 and analysed the data using a similar regression-based estimation approach to assess if, how, and when expectancy and association contribute to explain behavioral processing indices.

### Method

This study was conducted with the approval of the Deutsche Gesellschaft für Sprachwissenschaft (DGfS).

#### Participants

49 participants recruited through Prolific Academic Ltd. took part in the experiment, one of which was excluded due to inattentive reading (shown by short completion time and low accuracy). The remaining 48 participants (mean age 24.20; SD: 4.30; age range 18-32; 24 female) were all native speakers of German (8 early bilinguals) and had not indicated any language related disorders (such as reading difficulties). All participants gave their consent by agreeing to the written study conditions and were paid £6.25 for their participation.

#### Materials

The materials were the same as those used in Experiment 1.

#### Procedure

We conducted this experiment as a web-based study—as an ongoing pandemic prohibited in-lab experiments—using the software Ibex [76] and its PennController extension [91] (web based self-paced reading resulted in comparable reading time measures in a lab-to-web replication [92]). On each trial, participants were prompted to press the Enter-key to start reading, after which they were presented with a hash sign at the center of the screen indicating the position of the words. From then on, each word was presented centrally and participants had to press the Space-bar to proceed to the next word. On approximately one third of the trials, participants were presented with a comprehension question to which they had to respond using a *Yes* or *No* button (mapped to the *D* and *K* keys). The comprehension question could be about the content of any part of the experimental sentences to incentivize attentive reading of the entire sentence. We deem this a well-suited task for the web-based environment in which the experimenter can exert less control over the environment and behaviour of the participant, as task engagement with a comprehension-question should be larger than with binary plausibility judgements. The position of the answer options was reversed for half of the participants. We recorded participants’ response accuracy and decision time to the questions. After completion of ten practice trials, the materials were presented in three blocks of 80 trials each, half of which were fillers. After the practice and after each block, we provided coarse feedback on participants’ response accuracy. Participants were encouraged to take a short break between blocks. Due to technical limitations, the self-paced reading experiment differed from the EEG experiment in that words were presented in black font on white background.

#### Analysis

Analysis of reading times was conducted similarly to that of the ERP data: Cloze probabilities and association ratings were used as numerical predictors in linear mixed effects models that were then used to re-estimate the data. We analysed reading times on the word preceding the target (the *pre-critical region*), on the target word (the *critical region*) and, to capture spillover effects, on the two words following it (the *spillover* and *post-spillover region*). The spillover region always consisted of a closed class word (most commonly “und” / *and*), while the post-spillover region consisted of both closed and open class words. We considered each region as pertaining to a separate family of hypotheses. Hence, we did not correct for multiple comparisons across regions. Reading times were log-transformed to normalize their distribution. The Shapiro-Francia [93] test for normality, adequate for larger sample sizes, was however still significant on each region, suggesting non-normality.

### Results

#### Comprehension questions

Participants answered the comprehension questions correctly in 87.4% (SD = 33.2) of the experimental items (after data exclusion). Across the four conditions, accuracy was 90.2% in Condition A (A+E+; SD = 8.3), 88.4% in B (A–E+; SD = 11), 84.1% in C (A+E–; SD = 12.6), and 85.2% in D (A–E–; SD = 13). Average reaction times were 2538 ms in A (A+E+; SD = 670), 2458 ms in B (A–E+; SD = 589), 2829 ms in C (A+E–; SD = 838), and 2666 ms in D (A–E–; SD = 678). Means and standard deviations were computed from the per-subject and condition averages.

#### Reading times

**Fig 8:**
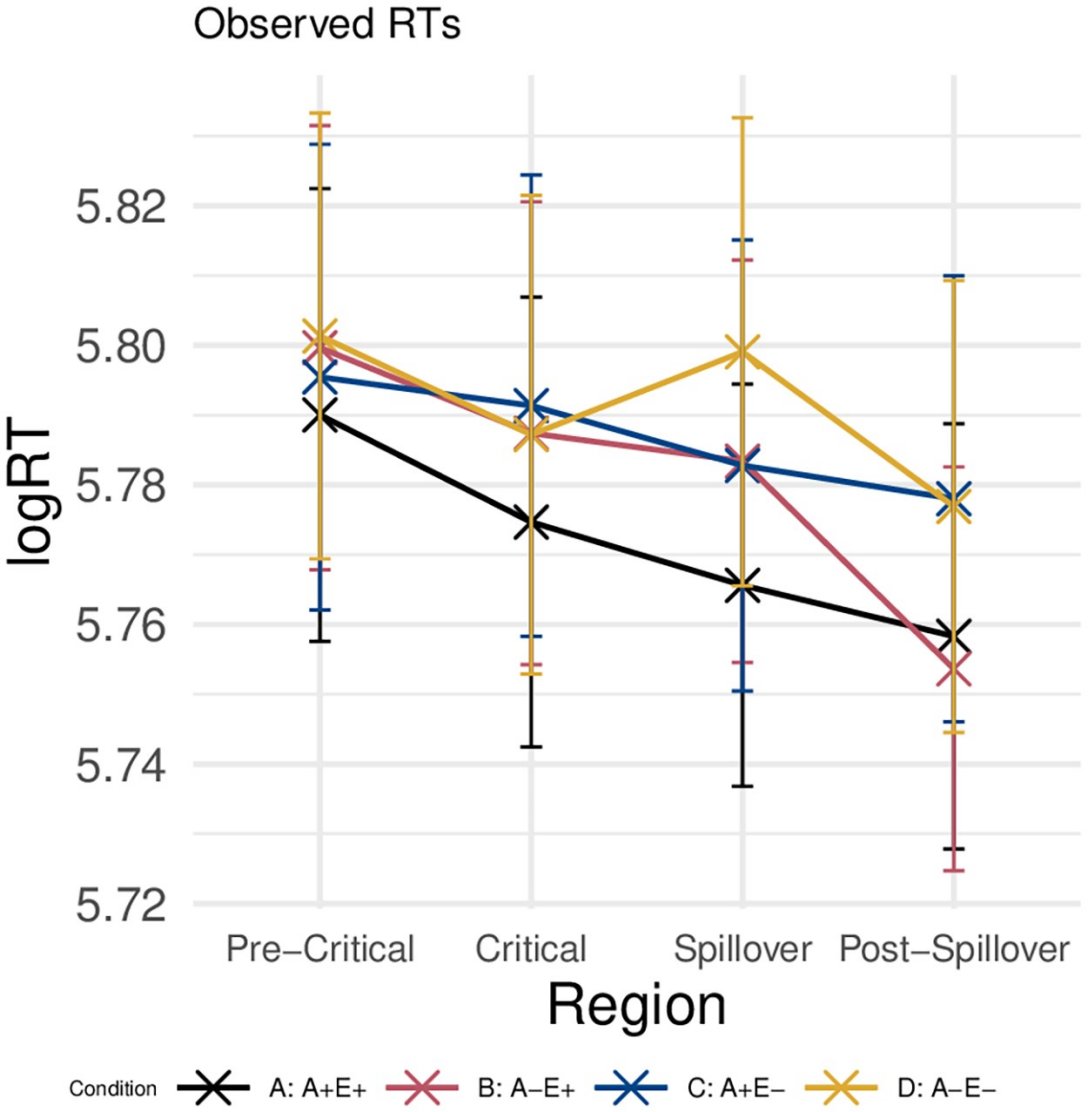
Reading times. Log Reading Times per condition on the pre-critical, critical, spillover, and post-spillover region. Error bars indicate standard error computed from the per-subject per-condition averages.

Fig 8 displays the average reading times on the pre-critical region (the article of the target word), the critical region (the target word, *axe*), the spillover region, and the post-spillover region. At each region, reading times are split up per condition. Reading times at the critical region slowed down for conditions B, C, and D. On the spillover region, association and expectancy had an additive effect, with slower reading times in the weakly associated (B and D) and unexpected (C and D) conditions relative to the baseline condition (A). Lastly, on the post-spillover region, association effects were no longer observed, and only trials in the unexpected conditions resulted in longer reading times.

As we did in the analysis of the ERP data, we first assessed whether transformations applied to the predictors led to improvements on the model residuals. We did not find any large differences between log-transformed and untransformed Cloze probability. With respect to the association ratings, we found that verb-target association did not account for log-transformed reading times over and above noun-target association (as it was with ERPs). In line with these findings, and in order to maximize comparability with the ERP results, we modelled log-transformed reading times as a linear function of log(Cloze) probability and noun-target association.

**Fig 9:**
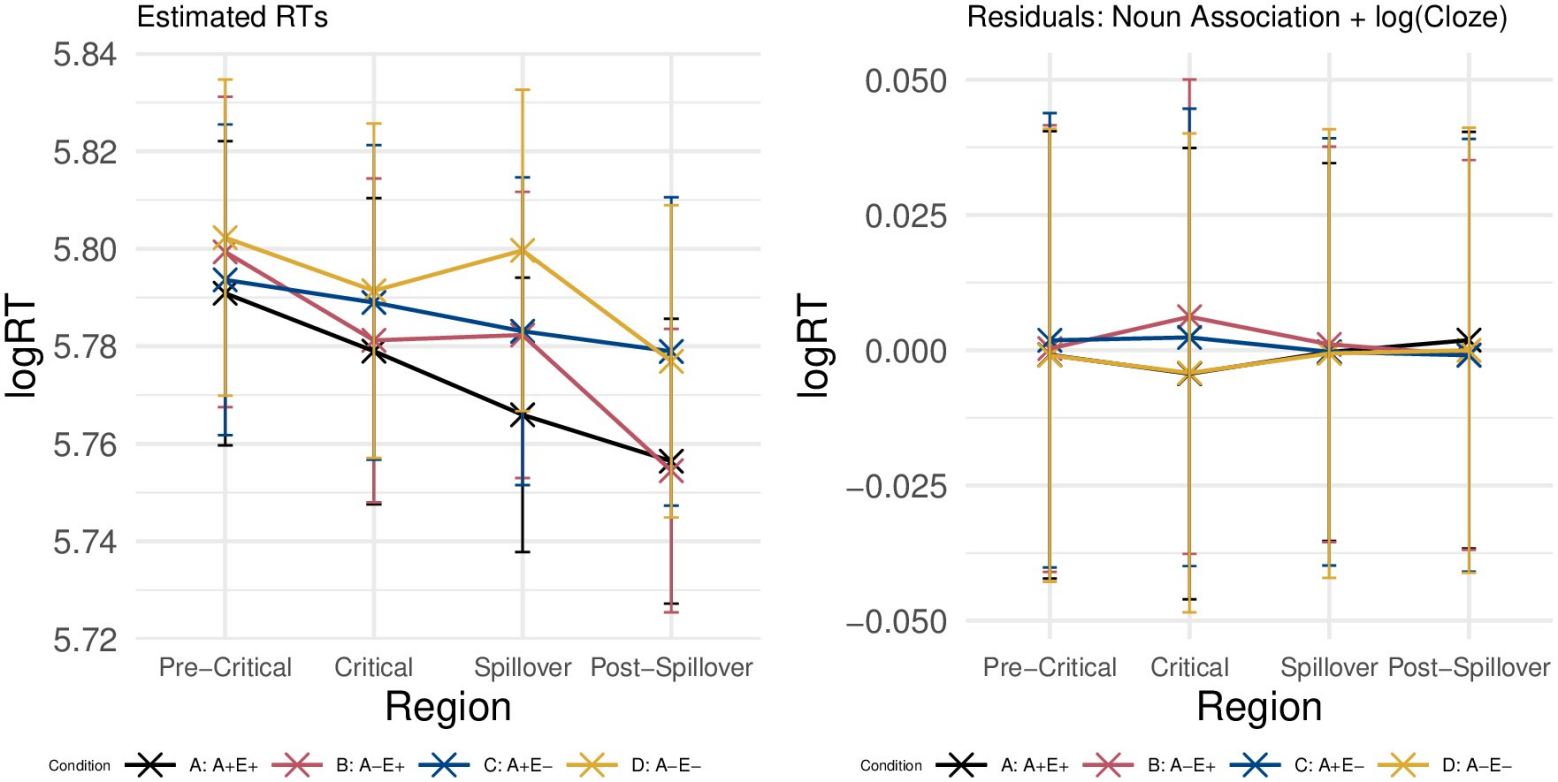
Estimated RTs and residual error. Estimated log-Reading Times (left) and residual error (right) per condition on the pre-critical, critical, spillover, and post-spillover region. Error bars indicate standard errors on the condition means.

The estimated reading times adequately model the observed reading times on the pre-critical, spillover, and post-spillover regions (as shown in Fig 9, left). This is not the case, however, in the critical region. This is due to the fact that, without an interaction term, the model is unable to arrive at a solution in which the estimated reading times of Condition B can be slowed without increasing the reading times of Condition D as well. This is reflected in the larger residual error for these two conditions in the critical region (Fig 9, right). Nevertheless, we decided to not include an interaction term in our models, as this complicates the interpretation of model coefficients.

**Fig 10:**
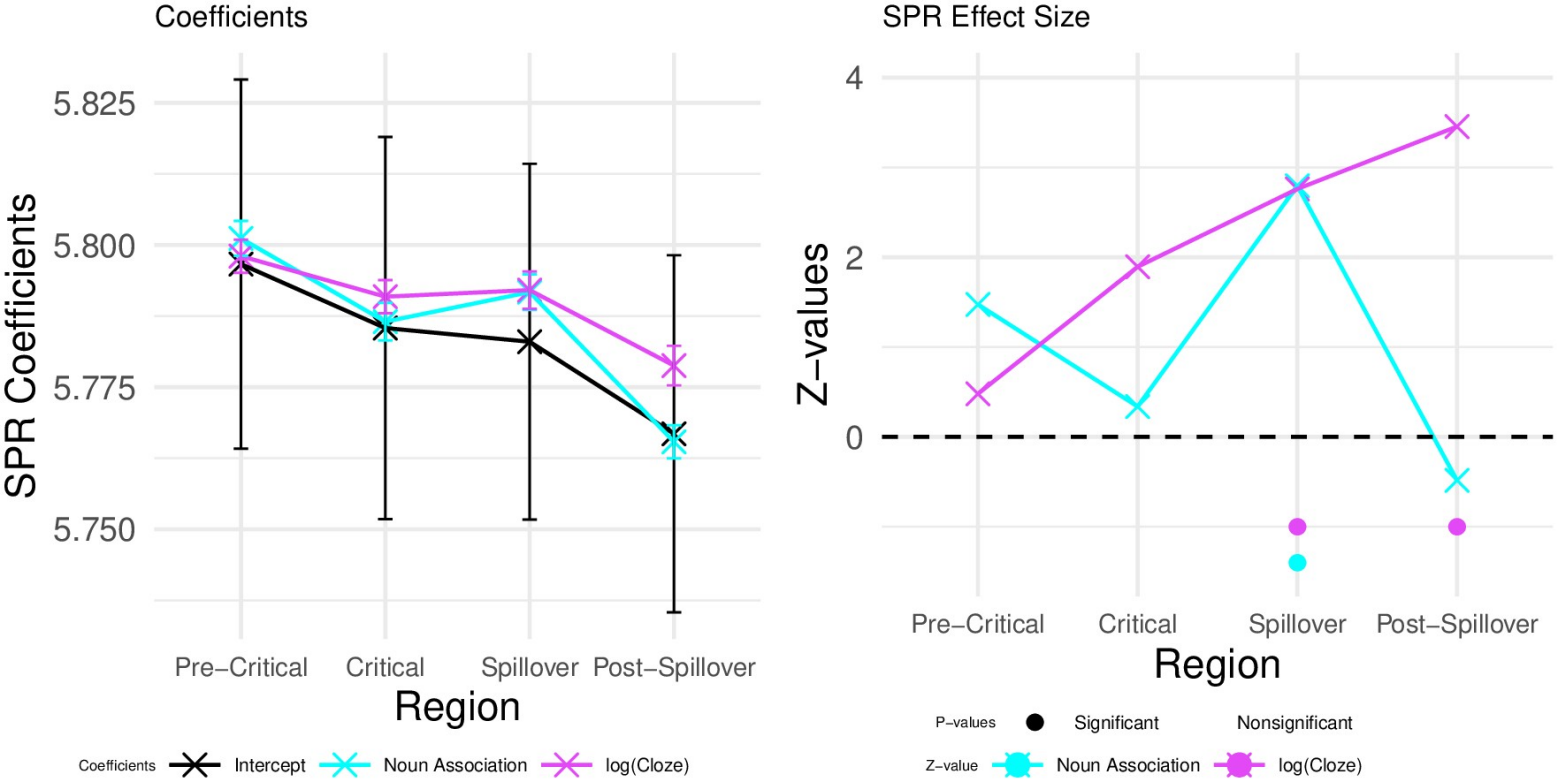
RT coefficients and z-values. Coefficicents (left, added to their intercept), effect sizes (z-values) and p-values (right) for each predictor on the pre-critical, critical, spillover, and post-spillover region. Error bars indicate the standard error on the coefficients from the statistical model.

The model coefficients and effect sizes in Fig 10 confirm the visual inspection of the reading times in each condition, as laid out above. There are no effects in the pre-critical region. Indeed, log(Cloze) alone accounts for increased reading times on the critical region, whereas on the spillover region log(Cloze) and noun-target association have an additive effect. On the post-spillover region, log(Cloze) alone predicts reading times departing from the intercept.

**Fig 11:**
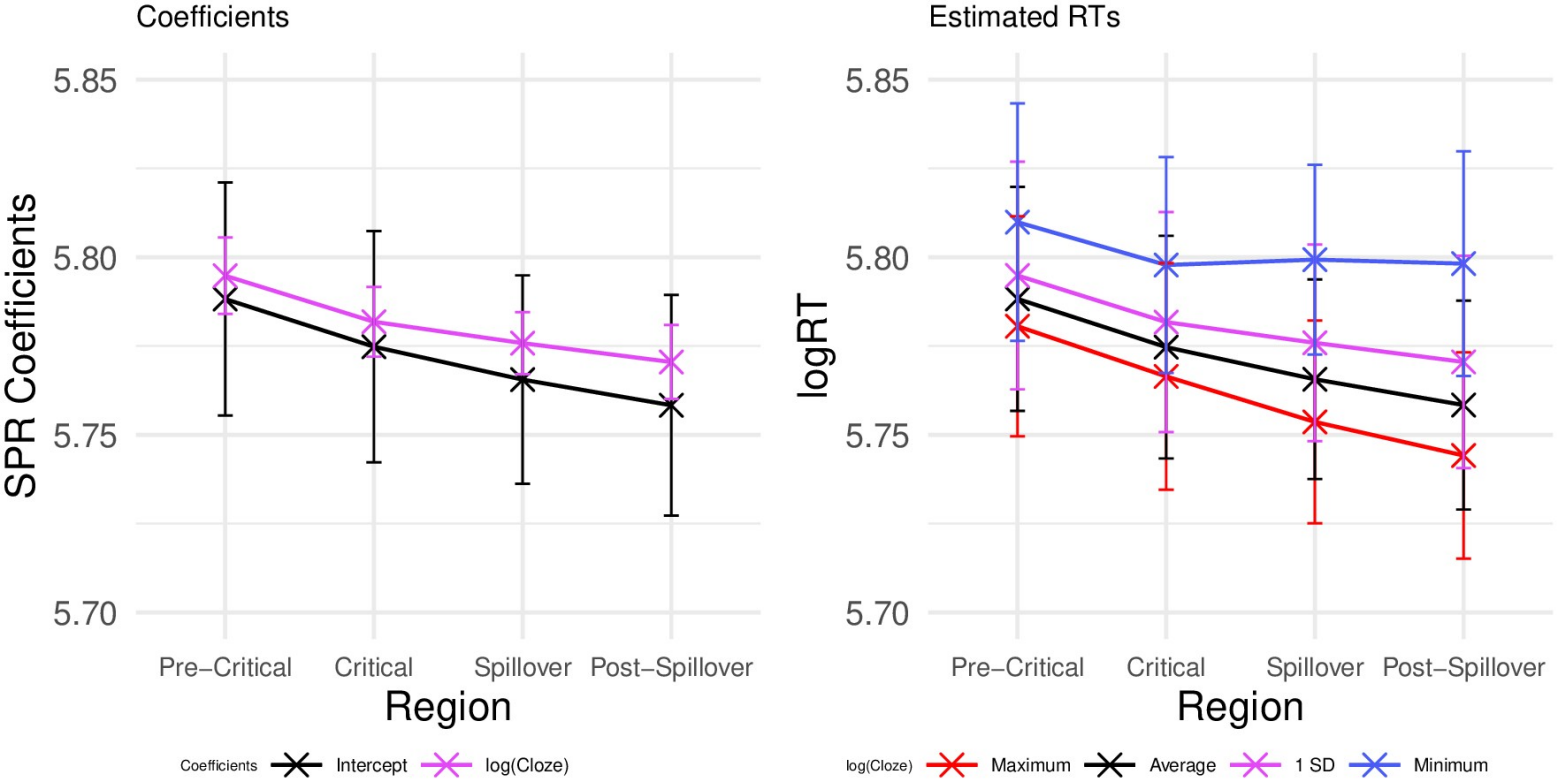
Exploratory RT analysis. Coefficients (left; added to their intercept) and estimated log-RTs (right) for exploratory LMER models fitted only on Condition A. Error bars indicate the standard error on the coefficients from the statistical model (right) and standard error computed from the per-subject per-value averages.

We also repeated the same analysis approach that was used in Experiment 1 to assess the graded effects of expectancy on the N400 and the P600 (see Section), i.e., we considered reading time data from trials in Condition A only. The results of this analysis are shown in Fig 11. Similarly to what we observed for the P600, log(Cloze) probability appears to have a graded effect on reading times, with increased reading times for lower Cloze-probability trials at the target and spillover regions in this post-hoc analysis.

### Discussion

In this self-paced reading study, we recorded reading times on exactly the same stimulus materials as in Experiment 1 to assess the relationship of association and expectancy in the behavioural domain and compare these results to the electrophysiological domain. Already on the critical region, reading times were slowed, albeit not significantly, for all manipulated conditions relative to the baseline. We observed that both expectancy and association influence reading times in the spillover region, while expectancy alone accounts for reading times on the word after it. This result is consistent with previous eye tracking findings showing robust effects of plausibility and short-living effects of association (e.g., [55]). Interestingly, the temporal distribution of the effects seems to align with the ERP patterns observed in Experiment 1. Both association and expectancy had an impact on somewhat earlier processing stages, that is, the N400 time window in Experiment 1 and the spillover word in Experiment 2. Expectancy alone had a later effect, corresponding to the P600 time window in Experiment 1 and the post-spillover region in Experiment 2. We return to this point in the General discussion. We also replicated the graded effect of expectancy in non-violating trials that we observed in Experiment 1. These findings provide further evidence that the processing effort observed in the present experiments does not merely index the detection of an anomaly, but rather reflects the degree to which a word (whether anomalous or not) is expected given the prior context.

## General discussion

We conducted two experiments aimed at disentangling the effects of expectancy and lexical association on electrophysiological (Experiment 1) and reading time (Experiment 2) measures of online processing, and examined if it is possible to identify a specific locus of expectancy effects in the ERP signal. In both experiments we tested sentences in which a direct object noun was either expected or unexpected given the selectional restrictions of the main verb (as measured through Cloze probability). Furthermore, the target was either highly or weakly associated with the content words of an adverbial clause preceding the target (as measured through lexical association norms). Critically, this adverbial clause was completely independent of the expectancy manipulation, avoiding any dependence between these often confounded factors. In sum, our design crossed the factors expectancy and association using a context manipulation.

The results of Experiment 1 reveal that the N400 component is sensitive to both expectancy and lexical association. Unexpected targets elicited a larger N400 amplitude than expected targets, and this effect was modulated by lexical association, with highly associated targets eliciting lower N400 amplitude than weakly associated ones. The P600, on the other hand, was sensitive to expectancy alone, with unexpected targets eliciting a larger P600 than expected ones. The results of Experiment 2 show that, while both expectancy and lexical association significantly influence reading times soon after the critical word, only expectancy has an effect downstream. The exploratory analysis conducted for both Experiment 1 and Experiment 2 provided preliminary evidence that the effect of expectancy is graded and does not depend on the presence of a semantic violation. In what follows, we discuss the main findings and their implications for neurocognitive accounts of language comprehension and the notion of Surprisal.

### The N400 is sensitive to both expectancy and lexical association

Both expectancy and lexical association contribute to predict the amplitude of the N400. This finding is consistent with a substantial body of evidence showing N400 effects of Cloze probability [17], word Surprisal [14, 16], and semantic similarity [79]. It is also consistent with several studies showing that N400 effects to semantic violations or implausibility are attenuated, or even overridden, when the eliciting word is semantically related to the context (e.g., [51, 60, 86, 94]). Interestingly, our design allowed us to establish that lexical association modulates the ERP signal also within semantically congruent items, as evidenced by the small N400 effect elicited by unrelated but expected targets relative to their related and expected counterparts. Taken together, these findings indicate that the N400 is sensitive to lexical association above and beyond expectancy. An important question is therefore to what extent the two effects hinge upon the same underlying cognitive mechanism as opposed to being qualitatively different.

We argue that the additive influences of these two properties can be naturally and parsimoniously accommodated within the memory-retrieval view of the N400 [13, 26–31]. On this view, the amplitude of the N400 reflects the ease with which the meaning of a word is accessed in long term memory. We define lexical access or retrieval as the cognitive process that maps perceived word forms onto its corresponding word meaning, taking context into account. This process is facilitated, among other factors, when this meaning is *associated* with conceptual knowledge activated by previous words in the context and/or when it can be *expected* given the unfolding utterance interpretation (see [45]). As a consequence, the retrieval account offers a parsimonious account of why both factors influence the N400.

### The P600 is sensitive to expectancy alone

In the P600 time window, we found that expectancy alone accounts for the positivity observed in centro-parietal sites. This effect can neither be explained in terms of syntactic processing difficulty, as our stimuli were syntactically well-formed and unambiguous, nor merely as a response to semantic violations (see [85]), as an exploratory analysis performed on a subset of data varying in Cloze probability suggested the continuous sensitivity of the P600 to expectancy in congruent trials. This result is thus consistent with a growing body of evidence indicating that the P600 is a general index of integration difficulty at different levels of analysis (e.g., [51, 62, 95–100]; see [26]). We define integration as the cognitive process that maps retrieved word meanings into the utterance meaning representation of the sentence so far, taking context into account. Under this interpretation, the effort involved in updating the unfolding utterance meaning is greater the more unexpected the utterance meaning resulting from integrating the meaning of the incoming word is.

Moreover, our data provide initial evidence that correlation may exist between Cloze and the amplitude of the P600, similar to to the established inverse correlation between the N400 and a word’s Cloze probability. Future work should further corroborate the gradedness of this link between the P600 and integration difficulty. Critically, however, studies aimed at assessing this relationship should control for spatiotemporal component overlap with the graded N400, resulting from retrieval (see [63] for discussion). That is, in order to obtain a clear view on the gradedness of the P600, overlap with the graded N400 should be factored out. Experimentally, this can effectively be achieved by strongly priming the target word while still varying its plausibility [62, 86]. Overall, the present findings provide compelling evidence that the P600 is a specific locus of expectancy effects, not sensitive to lexical association, consistent with the Retrieval-Integration account [26, 48].

### An integrated theory of the N400 and the P600

The functional interpretation of the N400 and P600 has been subject to debate for a long time. Based on the attenuation in N400 amplitude that we observed in response to associated adverbial clauses, we exclude the “pure” integration view of the N400 [23–25], which would predict an effect of expectancy alone. Similarly, it is our understanding that the computational model put forward by Rabovsky et al. [101], while capturing the expectancy effects, would not predict the association effect arising from the preceding adverbial clauses. These clauses were constructed so as to rule out any structural, or even semantically attractive (thematic) dependency with the target word, which is typically prerequisite for “good-enough” processing effects [65, 102]. The “hybrid” view of the N400 [32–34], however, can explain the observed N400 findings by assuming that both retrieval and integration processes are indexed by the N400. Nonetheless, the results are also completely aligned with a pure retrieval view of the N400 [13, 26–31] as well, under which both association and expectancy facilitate word retrieval.

The P600 in our data resulted from a violation of the main verb’s selectional restriction on its object, i.e. the target word. The resulting items were, however, syntactically well-formed, ruling out the view that the P600 serves as an index of morpho-syntactical processing [35–37] or syntactic integration [38, 39] alone. While conflict monitoring / resolution theories [40–44] could predict a P600 in response to the selectional restriction violation, such accounts generally have difficulty explaining biphasic N400-P600 patterns (see [26] for discussion) that are also present in our data. Lastly, the integration view of the P600 [26, 45] is completely in line with our results. Further, only the integration view would predict a graded sensitivity of the P600 to expectancy, as suggested by our post-hoc analysis.

In contrast to the Retrieval-Integration account, which combines the Retrieval view on the N400 and the Integration view on the P600, other theories typically focus on either the N400 or the P600, and therefore offer no account for their interdependence. Two notable exceptions, however, are the recent computational models by Rabovsky et al. [101] and Fitz & Chang [103]. While the former offers a computational instantiation of the N400 as integration, Rabovsky et al. [65] verbally theorise that P600 may reflect an attention-dependent revision process that can re-asses wrong interpretations generated by an automatic interpretation process indexed by the N400. Our design specifically avoids creating a semantic illusion that can be resolved by revision (see the Materials section) and, as such, the violation of expectancy should be reflected only in the N400 and not in the P600. Further, it is unclear how such attention-dependent revision processes would explain the graded P600 response to word expectancy suggested by our data. The model proposed by Fitz & Chang [103] successfully captures data from several ERP studies, and characterises the N400 and P600 as epiphenomena of error-based learning. The model accounts for the expectancy effect on the N400 as well as on the P600, with the latter being interpreted as a result of the selectional restriction violation. It is unclear, however, whether the model would predict the association effect from the adverbial clauses on the target word. Further, while this model predicts a graded link of the N400 to cloze probability, presumably, their model would also not predict a graded link of expectancy to the P600. Further enquiry into this latter point could thus provide strong test to dissociate between the RI model and those of Fitz & Chang [103] and Rabovsky et al. [65].

In sum, one would have to invoke several theories, explaining both ERP components individually in order to account for the entire ERP complex in our data. A key strength of the Retrieval-Integration account, on the other hand, is that it explains the entire ERP complex within one integrated-theory, making predictions for both components: The N400, as index of lexical retrieval, is sensitive to both lexical association and expectancy, whereas the P600, as index of integration, is sensitive to expectancy only.

### Dissociating retrieval and integration in behavioural measures

The results of Experiment 2 replicated the well-known effect of expectancy / Surprisal on reading times (e.g., [7, 9, 10, 12, 14, 48, 88]). An interesting question, however, is to what extent this behavioral cost reflects retrieval or integration processes (or both), as self-paced reading time is presumably the summation of several underlying processes. We observed that association and expectancy significantly predicted reading times on the spillover region, while the influence of expectancy remained up until the post-spillover region, suggesting that expectancy influences both early (retrieval) and later (integration) processes, similarly to what we observed with ERPs. An interesting open question is therefore how reading time effects in the time domain relate to ERP effects in the amplitude domain. The temporal dynamics of association and expectancy effects in reading times appears to echo the temporal pattern of the corresponding modulations in ERP components, with the N400 *effect* of association and expectancy preceding the P600 *effect* of expectancy alone (although the actual processes underlying the respective components do temporally overlap; see [62]). We can therefore speculate that the reading time increases in the spillover region capture a facilitation related to memory-retrieval for associated words, which also modulates the amplitude of the N400, while the cost in the post-spillover region reflects more demanding integrative processing, which in the electrophysiological domain is associated with increased P600 amplitude. Clearly, this is only speculative and would need to be examined in studies designed for this purpose. An experimental paradigm well-suited to address this issue could be one in which ERPs and self-paced reading times are recorded simultaneously (see [104–106]).

### The P600 is an index of comprehension-centric Surprisal

All contemporary models of language comprehension acknowledge the important role of expectancy in determining word processing difficulty. Among them, Surprisal theory [2, 4] posits that the cognitive effort incurred by each word in a sentence is proportional to its Surprisal, defined as the negative log-probability of a word given the prior context. Surprisal has been estimated using various language models (i.e., n-gram models, phrase-structure grammars, and recurrent neural networks), and has been shown to correlate with reading time [7–9] as well as N400 amplitude [16]. Interestingly, Frank et al. [16] interpret the N400 effect of Surprisal as supporting the memory-retrieval rather than the integration account of the N400, since retrieving lexical information associated with a word is predicted to be easier when the word is more predictable. The integration account was excluded based on the observation that Surprisal was estimated by language models that are only minimally (if at all) sensitive to semantics. Crucially, this may be the reason why Frank et al. [16] failed to find Surprisal effects on the P600 component (spatiotemporal component overlap being another possible explanation).

Rather than using language models, in the present study, expectancy was estimated using log-transformed Cloze probability (see [88]), which arguably more closely approximates a ‘comprehension-centric’, semantic notion of Surprisal that incorporates both linguistic experience and world knowledge [5]. The RI account predicts this notion of expectancy/Surprisal to influence both the N400 and the P600 component. First, Surprisal (and lexical association, among other factors) influences the ease with which the current word form is mapped to its word meaning (N400). Second, Surprisal influences the ease with which the current word meaning is integrated (P600) into the new, updated utterance meaning representation. Crucially, this integration view subsumes syntactically-, semantically-, and pragmatically-induced processing difficulties, as these may all hamper the construction of a coherent utterance meaning representation (see [26] for discussion).

In sum, in the neurocomputational model of incremental language comprehension proposed by Brouwer et al. [48], the comprehension-centric metric of Surprisal reflects the likelihood of an updated interpretation given the interpretation prior to integrating the meaning of the current word. Surprisal is thus predicted to be indexed by the P600 component, which reflects the effort involved in integrating the retrieved word meaning into the unfolding utterance interpretation: The more unexpected, unclear, or implausible the resulting utterance interpretation, the higher the amplitude of the P600.

## Conclusion

In this study, we investigated the contribution of expectancy and lexical association on ERP modulations and reading times, and whether a specific locus of expectancy-related effects can be established in the ERP signal. An ERP experiment revealed that the N400 is sensitive to both expectancy and lexical association while the P600 is sensitive only to expectancy. A post-hoc, exploratory, analysis suggests that the P600 is not only evoked in response to completely unexpected (zero Cloze) target words, but is also modulated by the degree of expectancy in non zero-Cloze targets. In a self-paced reading experiment, expectancy and lexical association influenced reading times on the spillover region, while the effect of expectancy extended into the post-spillover region. Based on the Retrieval-Integration account of the electrophysiology of language comprehension, we interpret the N400 and the P600 components to index two fundamental mechanisms involved in language comprehension, namely lexical retrieval and semantic integration, respectively. We further argue that word expectancy modulates neural and behavioural processing indices by facilitating / taxing both of these cognitive mechanisms.

On the one hand, the meaning of expected words as well as words that are strongly associated with the prior context is easier to retrieve from long term memory. On the other hand, unexpected words increase the effort involved in updating the unfolding utterance meaning representation with the retrieved word meaning.

Thus, while word expectancy influences both processes—retrieval and integration—they are qualitatively different processes that map different inputs to different outputs. Retrieval maps word forms into word meaning representations, while integration takes these word meanings and maps them into an updated utterance meaning representation. This view stresses that word expectancy effects are to be interpreted in terms of their consequences for cognitive processes, rather than as a process (e.g. that of anticipation) in and of itself. As the P600 was responsive to expectancy only, we argue that this component is the primary index of ‘comprehension-centric’ Surprisal, quantifying the difficulty incurred by integrating an incoming word’s meaning into the unfolding interpretation.
